# Diazoxide Attenuates Postresuscitation Brain Injury in a Rat Model of Asphyxial Cardiac Arrest by Opening Mitochondrial ATP-Sensitive Potassium Channels

**DOI:** 10.1155/2016/1253842

**Published:** 2016-08-28

**Authors:** Haidong Wu, Peng Wang, Yi Li, Manhui Wu, Jiali Lin, Zitong Huang

**Affiliations:** ^1^Department of Emergency Medicine, Sun Yat-sen Memorial Hospital, Sun Yat-sen University, Guangzhou, China; ^2^Institute of Cardiopulmonary Cerebral Resuscitation, Sun Yat-sen University, Guangzhou, China

## Abstract

*Objective*. We investigated whether and how diazoxide can attenuate brain injury after cardiopulmonary resuscitation (CPR) by selective opening of mitochondrial ATP-sensitive potassium (mitoKATP) channels.* Methods*. Adult male Sprague-Dawley rats with induced cerebral ischemia (*n* = 10 per group) received an intraperitoneal injection of 0.1% dimethyl sulfoxide (1 mL; vehicle group), diazoxide (10 mg/kg; DZ group), or diazoxide (10 mg/kg) plus 5-hydroxydecanoate (5 mg/kg; DZ + 5-HD group) 30 min after CPR. The control group (sham group, *n* = 5) underwent sham operation, without cardiac arrest. Mitochondrial respiratory control rate (RCR) was determined. Brain cell apoptosis was assessed using TUNEL staining. Expression of Bcl-2, Bax, and protein kinase C epsilon (PKC*ε*) in the cerebral cortex was determined by Western blotting and immunohistochemistry.* Results*. The neurological deficit scores (NDS) in the vehicle group decreased significantly at 24 h and 48 h after CPR. Diazoxide significantly improved NDS and mitochondrial RCR after CPR at both time points; 5-HD cotreatment abolished these effects. Diazoxide decreased TUNEL-positive cells following CPR, upregulated Bcl-2 and PKC*ε*, downregulated Bax, and increased the Bcl-2/Bax ratio; 5-HD cotreatment reversed these effects.* Conclusions*. Diazoxide attenuates postresuscitation brain injury, protects mitochondrial function, inhibits brain cell apoptosis, and activates the PKC pathway by opening mitoKATP channels.

## 1. Introduction

Brain injury is the major cause of disability and death after cardiac arrest and CPR. Ischemia-reperfusion injury (IRI) after cardiac arrest and CPR leads to brain injury, resulting in cerebral cell necrosis, apoptosis, and oxidative stress injury. The brain is particularly vulnerable to IRI due to the specific structural and functional characteristics of brain tissue.

Although therapeutic hypothermia has been recommended for comatose survivors to improve neurologic outcomes following cardiac arrest, the efficacy of this treatment remains controversial, especially in children [1, 2]. Therefore, alternative approaches are required to further improve neurologic outcomes in patients following cardiac arrest.

Following cardiac arrest and CPR, mitochondria play a critical role in producing and eliminating free radicals and in mediating oxidative stress injury. The pathophysiological mechanisms of brain damage after CPR are related to secondary mitochondrial injury and apoptosis; thus, therapeutic strategies aimed at preventing IRI-induced mitochondrial injury and apoptosis may improve functional recovery of the brain [3].

Ischemic preconditioning and postconditioning protect cells against IRI [4]. The mitochondrial ATP-sensitive potassium (mitoKATP) channel is important for protecting cells and organs from injury during ischemic preconditioning and postconditioning [5]. Recent evidence has suggested that the selective mitoKATP channel agonist diazoxide potently reduces mitochondrial injury by preserving mitochondrial integrity and inhibiting apoptosis in neurons [6, 7]. Thus, targeting the mitoKATP channel may represent a novel approach to therapeutic strategies for neuroprotection [8].

Considering the serious consequences and poor prognosis of brain injury after cardiac arrest and CPR, we investigated the effects of diazoxide-mediated opening of mitoKATP channels on postresuscitation brain injury and aimed to elucidate the neuroprotective mechanisms of diazoxide in a rat model of cardiac arrest.

## 2. Methods

The study protocol was approved by the Sun Yat-sen University Animal Experimentation Ethics Committee (number SCXK2013-07-0028) and met the Guidelines for Ethical Conduct in the Care and Use of Experimental Animals published by the Ministry of Science and Technology of the People's Republic of China.

### 2.1. Animal Preparation

Male Sprague-Dawley rats (Experimental Animal Center of Sun Yat-Sen University, Grade II, Certification number SCXK (YUE) 2013-0029) weighing 300–400 g were fasted overnight, with free access to water. Global cerebral ischemia and reperfusion injuries were induced by asphyxial cardiac arrest followed by CPR. Rats were anesthetized by intraperitoneal injection of pentobarbital sodium (45 mg/kg body weight; Sigma, St. Louis, MO), with additional doses (10 mg/kg) added at intervals of approximately 1 h, as necessary. Animals were orally intubated with a 14-gauge cannula (Abbocath-T, Hospira, Lake Forest, IL) into the trachea for ventilation.

We advanced a 23-gauge polyethylene 50 (PE-50) catheter (Abbocath-T) into the right external jugular vein for intravenous infusion and another into the thoracic aorta from the left femoral artery for measurement of mean arterial pressure (MAP). MAP was measured with a pressure transducer (BD, Franklin Lakes, NJ). Before insertion, the catheters were filled with physiological salt solution containing 5 IU/mL heparin to prevent blood coagulation. We maintained the rats' rectal temperature at 36.5 ± 0.5°C using a heating lamp. Hemodynamic data and electrocardiograms were monitored with a multifunctional physiological monitor throughout the procedure (Spacelab Healthcare, Snoqualmie, WA).

### 2.2. Experimental Protocol

After baseline measurements were obtained, anesthetized rats were paralyzed with vecuronium bromide 2 mg/kg (Xianjupharmacy, Zhejiang, China). Asphyxia and cardiac arrest were induced by clamping the endotracheal tube according to the method described in our previous report [9]. Approximately 4 minutes following induction of apnea, cardiac arrest was determined by loss of aortic pulse and MAP ≤ 20 mmHg. Six minutes after the onset of cardiac arrest, precordial compression was initiated with an electrically driven mechanical chest compressor, while mechanical ventilation was initiated with pure oxygen (FiO_2_ 100%) at a tidal volume of 6 mL/kg and a frequency of 50 breaths/min. The precordial compression rate was maintained at 250/min and was synchronized with a compression/ventilation ratio of 5 : 1, with equal compression-relaxation duration. We maintained arterial diastolic pressure at 25 ± 5 mmHg by adjusting the depth of compression. A 4-J biphasic waveform electrical shock (M-Series, Zoll Medical Corporation; Chelmsford, MA) was used to stop ventricular fibrillation (VF) if VF occurred after 4 min of CPR. Adrenaline (20 *μ*g/kg) was intravenously injected after 2 min of CPR. Return of spontaneous circulation (ROSC) was defined as return of a supraventricular rhythm with MAP > 60 mmHg, lasting for approximately 5 min. The animals underwent intensive care with mechanical ventilation for 1 h after ROSC. Animals were extubated and placed in their cages after their upper airway reflexes became active.

### 2.3. Experimental Design

Thirty minutes after ROSC, rats were randomized into the vehicle-treated (vehicle), diazoxide-treated (DZ), or diazoxide + 5-hydroxydecanoate- (5-HD-) treated (DZ + 5-HD) groups (*n* = 10 each). Vehicle group animals received 1 mL of 0.1% dimethyl sulfoxide (DMSO) intraperitoneally; DZ group animals received 10 mg/kg diazoxide intraperitoneally, while DZ + 5-HD group animals received 10 mg/kg diazoxide plus 5 mg/kg 5-HD intraperitoneally. Diazoxide (Sigma-Aldrich) was dissolved in 0.1% DMSO for injection, while 5-HD (Sigma-Aldrich) was dissolved in saline. A normal control group (sham group, *n* = 5) also underwent identical anesthetic and surgical procedures without the induction of cardiac arrest.

### 2.4. Neurological Functional Testing

Functional neurological testing of the rats was performed 24 h and 48 h after ROSC by an observer blinded to experimental conditions. Neurological deficit scores (NDS) were digitalized on a scale of 0–80 [10], based on a composite of arousal, reflex, motor, sensory, and balance responses, with 0 corresponding to brain death and 80 to no deficit.

### 2.5. Detection of Mitochondrial RCR

Half of the surviving animals in all groups were sacrificed by carbon dioxide asphyxiation 24 h after ROSC, and their brain tissues were removed for subsequent examinations. After rats were sacrificed, cortical tissues were rapidly separated, weighed, and placed in an ice-cold Dounce homogenizer. We isolated brain mitochondria as previously reported [11]. A Clark oxygen electrode system (OxygraphTM, Hansatech Instruments, King's Lynn, UK) was used to determine mitochondrial respiratory function. Reaction buffer (2.5 mL, consisting of 125 mM potassium chloride, 2.5 mM KH_2_PO_4_, 20 mM HEPES, 4 mM magnesium chloride, 0.1% BSA, and 225 mM mannitol) was stirred to a steady state in a sealed reaction tank at 25°C, pH 7.4. After stabilization of the recorded curve, 2.5 mL of the mitochondrial suspension was added, and samples were incubated for 1 min. We then added 20 *μ*L disodium succinate (4 mM) to the tank, following which the oxygen concentration slowly declined, reflecting respiratory state 4 (R4). Next, we added 20 *μ*L of adenosine diphosphate (ADP, 50 mM), after which the oxygen concentration rapidly declined, reflecting respiratory state 3 (R3). The R3/R4 ratio reflected the respiratory control rate (RCR), which is a measure of the integrity of the mitochondrial membrane and oxidative phosphorylation: decreased RCR suggests impaired mitochondrial function.

### 2.6. TUNEL Staining

In order to quantify the rate of apoptosis, a terminal deoxynucleotidyl transferase-mediated dUTP nick-end labeling (TUNEL) assay was performed on paraffin-embedded tissue sections of each animal. We performed TUNEL staining according to the manufacturer's instructions (Roche Molecular Biochemical, Mannheim, Germany) in order to identify apoptotic cells in paraffin sections of rat brains. We counted cells with nuclear brown-stained particles as TUNEL-positive in five randomly selected high-power fields (400x) on each slide. The apoptosis index (AI) in each group was averaged from the values determined using the following formula: AI = (number of apoptotic cells/total number of cerebral cells) × 100%.

### 2.7. Western Blotting Analysis

Expression of Bcl-2, Bax, and PKC*ε* protein in the rat cortex was detected using Western blotting. The frozen tissue samples were completely homogenized in RIPA lysis buffer (Sigma-Aldrich) containing protease inhibitors. Lysates were clarified by centrifugation at (10,800 ×g) for 15 min at 4°C, and the lysate proteins were separated by sodium dodecyl sulfate polyacrylamide gel electrophoresis. We transferred the proteins to polyvinylidene fluoride membranes (Millipore, Billerica, MA) and blocked the membranes overnight (5% milk powder in Tris-buffered saline). The membranes were incubated with mouse anti-PKC*ε* primary antibody (Abcam, Cambridge, MA), mouse anti-Bcl-2 primary antibody (Abcam), or mouse anti-Bax primary antibody (Abcam). Bands were visualized using the ECL Western Blotting Substrate Kit (Pierce, Rockford, IL). After scanning the blots, the intensity of the bands was determined using Image J version 7.0 (National Institutes of Health, Bethesda, MD) densitometry software. The resulting values were normalized against Glyceraldehyde 3-phosphate dehydrogenase (GAPDH) expression as an internal control. We performed a minimum of 3 blots for each protein analysis.

### 2.8. Immunohistochemistry Analysis

For immunohistochemistry, we deparaffinized the sections, which were subsequently washed three times in phosphate-buffered saline (PBS) for 5 min. The sections were then blocked with 5% serum for 30 min. Slides were incubated overnight with primary antibodies against PKC*ε*, Bcl-2, or Bax (Abcam) at 4°C. After rinsing three times with PBS, slides were incubated with secondary antibodies at 37°C for 20–30 min, followed by incubation with 3,30-diaminobenzidine tetrahydrochloride solution and counterstaining with hematoxylin. Five different high-power fields per slide were observed by light microscopy.

### 2.9. Statistical Analysis

All data are presented as mean ± standard deviation (SD). Statistical power analyses were performed using SPSS version 19.0 (IBM Analytics, Chicago, IL, United States) for Windows. Unpaired *t*-tests were used to compare the parameters between two groups, while the Newman-Keuls test was used for multiple group comparisons. *P* < 0.05 was considered statistically significant.

## 3. Results

### 3.1. Diazoxide Improves Neurological Outcomes

**Figure 1:**
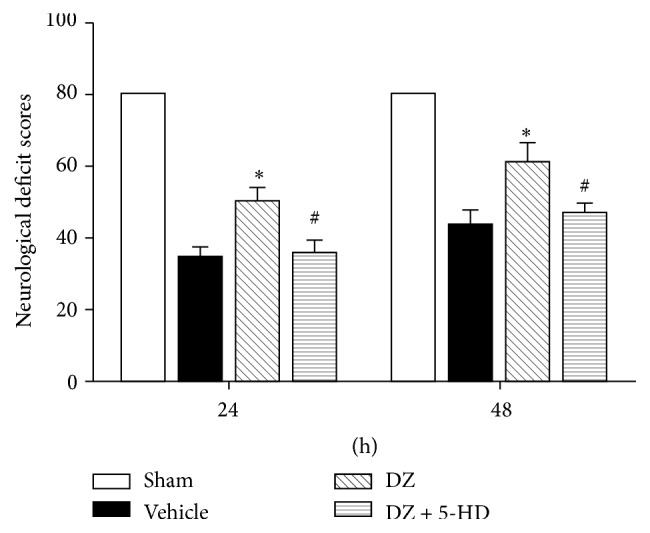
Neurological deficit scores for rats in the sham, vehicle group, DZ group, and DZ + 5-HD group at 24 h and 48 h after ROSC. Data are presented as means ± SD, *n* = 5 rats/group. ^*∗*^ *P* < 0.05 versus vehicle group; ^#^ *P* < 0.05 versus DZ group. 5-HD: 5-hydroxydecanoate; DZ: diazoxide; ROSC: return of spontaneous circulation.

All sham-operated animals exhibited normal NDS at both time points (NDS: 80); however, NDS in the vehicle group decreased significantly at 24 h and 48 h after CPR. NDS improved significantly at 24 h and 48 h after CPR following diazoxide treatment, though this effect was abolished by cotreatment with 5-HD (Figure 1).

### 3.2. Diazoxide Improved Mitochondrial RCR

Mitochondrial RCR reflects the efficiency of oxidative phosphorylation as well as mitochondrial function. R3 and mitochondrial RCR of the vehicle group were significantly lower than those of the sham group (*P* < 0.05), while R3 and mitochondrial RCR of the DZ group were significantly higher than those of both the vehicle group and the DZ + 5-HD group (*P* < 0.05) 24 h after ROSC (see Supplementary Figure 1 in Supplementary Material available online at http://dx.doi.org/10.1155/2016/1253842). No significant differences in R4 were observed among the four groups at 24 h after ROSC. These results suggest that diazoxide protects mitochondrial respiratory function in rat brain cells after ROSC, but this effect can be abolished by cotreatment with 5-HD.

### 3.3. Diazoxide Inhibits Apoptosis in the Brain after CPR

TUNEL-positive cells in the cortical regions were scored as apoptotic. In the sham group, the percentage of TUNEL-positive cells was markedly low (see Supplementary Figure 2). In the vehicle group; however, the percentage of TUNEL-positive cells increased following CPR when compared to that of the sham group (*P* < 0.05). In the DZ group, the percentage of TUNEL-positive cells decreased significantly following CPR when compared to that of the vehicle group (*P* < 0.05). This effect was abolished by cotreatment with 5-HD (DZ versus DZ + 5-HD groups, *P* < 0.05).

### 3.4. Diazoxide Increases the Bcl-2/Bax Ratio

Bcl-2 protein expression decreased significantly in the vehicle group following ROSC when compared to that of the sham group. Following administration of diazoxide, Bcl-2 protein expression increased significantly after ROSC when compared with that of the vehicle group, though this effect was negated by cotreatment with 5-HD. In contrast, Bax protein expression increased significantly following ROSC in the vehicle group when compared to that of the sham group. Compared to the vehicle group, Bax protein expression decreased significantly after administration of diazoxide in the DZ group; again, this effect was reversed upon cotreatment with 5-HD. Changes in the Bcl-2/Bax ratio also remained consistent with these variations (see Supplementary Figures 2 and 3).

### 3.5. Diazoxide Increases PKC*ε* Expression

PKC*ε* protein expression decreased significantly following ROSC in the vehicle group when compared to that of the sham group. After administration of diazoxide, PKC*ε* protein expression increased significantly following ROSC when compared to that of the vehicle group. This effect was again abolished by cotreatment with 5-HD (see Supplementary Figure 4).

## 4. Discussion

The results of the present study indicate that administration of diazoxide following global cerebral ischemia/reperfusion induced by cardiac arrest/CPR may protect mitochondrial respiration and energy synthesis, inhibit apoptosis, and improve neurological function by opening mitoKATP channels. These results are consistent with those of previous studies that have reported the neuroprotective effects of diazoxide [7, 8].

Diazoxide preconditioning has been proven effective for the treatment of focal brain injuries [12–14]; however, preconditioning is infeasible for global brain injuries following unpredictable experiences of cardiac arrest. A previous study has observed that diazoxide postconditioning can attenuate ischemia/reperfusion-induced injuries in the rat liver [15] and induce mitochondrial protein S-nitrosylation as well as redox-sensitive mitochondrial phosphorylation/translocation of RISK elements [16]. The results of the present study also demonstrate that diazoxide postconditioning may be a potential therapeutic strategy for the treatment of global brain injury following cardiac arrest.

The pathophysiologic mechanisms of brain damage secondary to cardiac arrest and CPR include temporary global cerebral ischemia and reperfusion injury, impairment of mitochondrial energy functions, and secondary apoptosis during and after CPR [17]. Some studies have reported that postischemic inflammatory responses and apoptosis are involved in brain damage after cerebral ischemia [18]. Interventions that can reduce secondary brain injury may improve survival and neurological recovery, though most brain injuries cannot be rapidly treated following cardiac arrest and CPR, and the cascade of inflammatory responses and apoptosis may be inevitable; effective measures that can prevent brain injury after CPR remain limited.

Inhibition of postresuscitation mitochondrial apoptotic executioners may also provide considerable neuroprotection [3]. Some studies have shown that opening of the mitoKATP channel causes mitochondrial membrane depolarization, decreased mitochondrial membrane potential, and increased mitochondrial volume and that this has antiapoptotic effects, resulting in PKC activation and inhibition of reactive oxygen species (ROS) production during ischemia [19]. When taken with these previous findings, the results of the present study indicate that diazoxide, which opens mitoKATP channels, may attenuate cerebral injuries induced by hypoperfusion or ischemia/reperfusion [6, 7].

Mitochondria, the energy-producing power stations of the cell, play a critical role as effectors and targets of ischemia/reperfusion injury following cardiac arrest. Mitochondria are involved in oxidative stress, calcium overload, and apoptosis cascades that occur during IRI after CPR. Previously, our group [20] and others [21] have demonstrated that myocardial mitochondrial function is impaired and that mitochondrial ultrastructure changes occur during cardiac arrest and following ROSC. Further research has indicated that cerebral mitochondrial oxidative phosphorylation is also impaired following CPR in a rat model of ventricular fibrillation [11]. Xu et al. observed that RCR decreases after resuscitation in a rat model of KCl-induced cardiac arrest [22]. However, little is known regarding the dysfunction of cerebral mitochondrial during cardiac arrest and following ROSC. In the present study, we demonstrated that mitochondrial R3 and RCR decreased significantly following ROSC and that this decrease is significantly attenuated by diazoxide. Furthermore, the effect of diazoxide was abolished by 5-HD, a specific antagonist of the mitoKATP channel. Therefore, diazoxide may be a potential drug for improving cerebral mitochondrial function after CPR via opening of mitoKATP channels.

Apoptosis after cardiac arrest is another important cause of neuronal degeneration, leading to neurological dysfunction after global cerebral ischemia [23]. Interactions between the proapoptotic and antiapoptotic proteins of the Bcl-2 family on the outer mitochondrial membrane are important in apoptosis. Upregulation of Bcl-2 or downregulation of Bax has been found to attenuate apoptosis; hence, an increased Bcl-2/Bax protein ratio inhibits neuronal apoptosis [3]. However, effective approaches for preventing or limiting neuronal damage due to apoptosis after cardiac arrest remain elusive. Recently, researchers have observed that diazoxide attenuates graft injury and suppresses hepatic ischemia/reperfusion injury after mouse liver transplantation via a Bcl-2-dependent mechanism [24]. In the present study, we revealed that diazoxide enhances Bcl-2 expression and inhibits Bax expression, thereby increasing the Bcl-2/Bax ratio and inhibiting apoptosis following CPR. Altogether, these results suggest that diazoxide may suppress apoptosis that occurs in conjunction with postresuscitation global cerebral ischemia and may contribute to neurological functional recovery by opening mitoKATP channels.

PKC regulates key cytoprotective mitochondrial functions, including electron transport chain activity, ROS generation, mitochondrial permeability transition, and detoxification of reactive aldehydes [25]. Activation of PKC also protects mitochondria by activating mitoKATP channels [26], while downregulation of PKC inhibits diazoxide-induced activation of mitoKATP channels [27]. Activation of mitoKATP channels for cardiac protection against ischemic injury is dependent on PKC activity [28]. PKC*ε* is an isoform of PKC that regulates mitochondrial pools of nicotinamide adenine dinucleotide (NAD) or nicotinamide phosphoribosyl transferase and affects ischemic preconditioning [29]. In a study of myocytes, researchers observed that opening of mitoKATP channels may activate PKC*ε* and induce its translocation into myofibrillar-like structures. PKC*ε* activation occurs downstream of the mitoKATP channel, possibly as a result of ROS production after the opening of mitoKATP channels [30]. In the present study, we demonstrated that diazoxide can increase PKC*ε* expression but that this increase in expression is abrogated by 5-HD. The neuroprotective effect of diazoxide after CPR may occur via activation of the PKC pathway after opening of mitoKATP channels.

The present study possesses some limitations. As this is a rat model, care should be exercised when extrapolating the results to human patients. Further research utilizing animal models more similar to humans with respect to cardiac arrest and CPR is required prior to clinical studies of the neuroprotective effects of diazoxide following CPR.

## 5. Conclusion

In conclusion, the results of the present study indicate that diazoxide-induced opening of mitoKATP channels attenuates postresuscitation brain injury and protects against impaired mitochondrial function, modulates the expression of apoptotic proteins, inhibits cell apoptosis, and activates the protective PKC signaling pathway. Our findings provide new insight into strategies for improving the therapeutic efficacy of treatments for postresuscitation brain injury. However, the precise mechanisms underlying the opening of mitoKATP channels and neuroprotective activity of PKC remain to be elucidated.

## Supplementary Material


**Supplementary Figure 1. **Comparison of cerebral cell mitochondrial respiratory parameters in different groups 24 h after ROSC. Data are presented as means ± SD, n = 5 rats/group. ∗*P < *0.05 vs. sham group, ^#^ *P < *0.05 vs. vehicle group, ∗∗*P < *0.05 vs. DZ group. 5-HD: 5-hydroxydecanoate; DZ: diazoxide; RCR: respiratory control rate.
**Supplementary Figure 2. **TUNEL staining analysis following ROSC
A: Quantitative analysis of TUNEL-positive cells in cortical regions 24 h after ROSC in the four rat groups. Five randomly-selected high-power (× 400) fields were analyzed per section, and the percentage of positive cells (positive cells/total cells × 100%) was calculated to determine the apoptotic index. Data are presented as means ± SD, n = 5 rats/group. ∗*P < *0.01 vs. sham group; ^#^ *P < *0.05 vs. vehicle group; ∗∗*P < *0.05 vs. DZ group
B: Representative photomicrographs of TUNEL staining. 5-HD: 5-hydroxydecanoate; DZ: diazoxide. ROSC: return of spontaneous circulation
**Supplementary Figure 3. **Expression of Bcl-2, Bax, and PKCε protein following ROSC. Expression of Bcl-2, Bax, and PKCε protein in the cerebral cortex 24 h after ROSC in a rat model of cardiac arrest was assessed using Western blotting (A). Western blots were quantified by densitometry (BE). Data are presented as means ± SD, n = 5 rats/group. ∗*P < *0.01 vs. sham group; ^#^ *P < *0.05 vs. vehicle group; ∗∗*P < *0.05 vs. DZ group; ROSC: return of spontaneous circulation; GAPDH: Glyceraldehyde 3-phosphate dehydrogenase.
**Supplementary Figure 4. **Expression of Bcl-2, Bax, and PKCε protein in the cerebral cortex 24 h after ROSC in the four rat groups obtained via immunohistochemical staining. (A, E, I) Sham; (B, F, J) Vehicle; (C, G, K) DZ; (D, H, L) DZ+5-HD; ROSC: return of spontaneous circulation.
