# Supplementary material for: Diazoxide Attenuates Postresuscitation Brain Injury in a Rat Model of Asphyxial Cardiac Arrest by Opening Mitochondrial ATP-Sensitive Potassium Channels
Source: Biomed Res Int. 2016 Aug 28;2016:1253842. doi: 10.1155/2016/1253842 (PMC5018309; doi:10.1155/2016/1253842)
Supplement: Supplementary file 1 — Supplementary Figure 1. Comparison of cerebral cell mitochondrial respiratory parameters in different groups 24 h after ROSC. Data are presented as means ± SD, n = 5 rats/group. ∗P < 0.05 vs. sham group, # P < 0.05 vs. vehicle group, ∗∗P < 0.05 vs. DZ group. 5-HD: 5-hydroxydecanoate; DZ: diazoxide; RCR: respiratory control rate. Supplementary Figure 2. TUNEL staining analysis following ROSC A: Quantitative analysis of TUNEL-positive cells in cortical regions 24 h after ROSC in the four rat groups. Five randomly-selected high-power (× 400) fields were analyzed per section, and the percentage of positive cells (positive cells/total cells × 100%) was calculated to determine the apoptotic index. Data are presented as means ± SD, n = 5 rats/group. ∗P < 0.01 vs. sham group; # P < 0.05 vs. vehicle group; ∗∗P < 0.05 vs. DZ group B: Representative photomicrographs of TUNEL staining. 5-HD: 5-hydroxydecanoate; DZ: diazoxide. ROSC: return of spontaneous circulation Supplementary Figure 3. Expression of Bcl-2, Bax, and PKCε protein following ROSC. Expression of Bcl-2, Bax, and PKCε protein in the cerebral cortex 24 h after ROSC in a rat model of cardiac arrest was assessed using Western blotting (A). Western blots were quantified by densitometry (BE). Data are presented as means ± SD, n = 5 rats/group. ∗P < 0.01 vs. sham group; # P < 0.05 vs. vehicle group; ∗∗P < 0.05 vs. DZ group; ROSC: return of spontaneous circulation; GAPDH: Glyceraldehyde 3-phosphate dehydrogenase. Supplementary Figure 4. Expression of Bcl-2, Bax, and PKCε protein in the cerebral cortex 24 h after ROSC in the four rat groups obtained via immunohistochemical staining. (A, E, I) Sham; (B, F, J) Vehicle; (C, G, K) DZ; (D, H, L) DZ+5-HD; ROSC: return of spontaneous circulation. [file 1253842.f1.docx]

**Supplemental Figures**


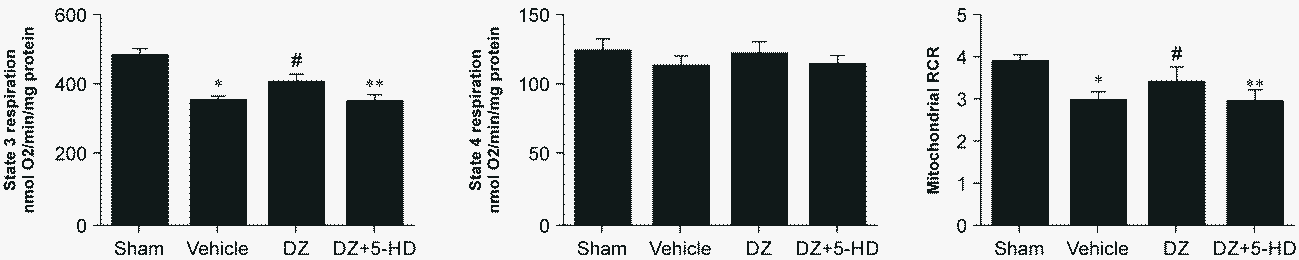


**Supplementary Figure 1.** Comparison of cerebral cell mitochondrial respiratory parameters in different groups 24 h after ROSC. Data are presented as means ± SD, n = 5 rats/group. **P <* 0.05 vs. sham group, ^#^*P <* 0.05 vs. vehicle group, ***P <* 0.05 vs. DZ group. 5-HD: 5-hydroxydecanoate; DZ: diazoxide; RCR: respiratory control rate.


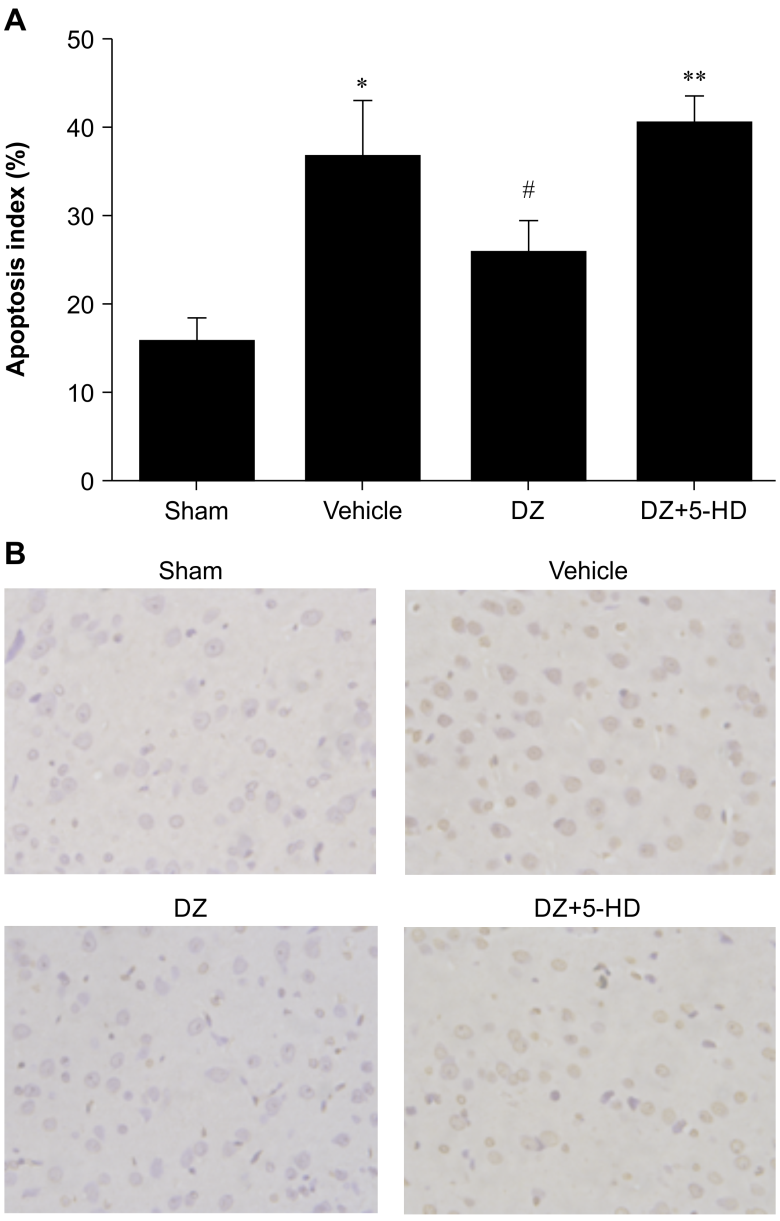


**Supplementary Figure 2.** TUNEL staining analysis following ROSC

A: Quantitative analysis of TUNEL-positive cells in cortical regions 24 h after ROSC in the four rat groups. Five randomly-selected high-power (× 400) fields were analyzed per section, and the percentage of positive cells (positive cells/total cells × 100%) was calculated to determine the apoptotic index. Data are presented as means ± SD, n = 5 rats/group. **P <* 0.01 vs. sham group; ^#^*P <* 0.05 vs. vehicle group; ***P <* 0.05 vs. DZ group

B: Representative photomicrographs of TUNEL staining. 5-HD: 5-hydroxydecanoate; DZ: diazoxide. ROSC: return of spontaneous circulation


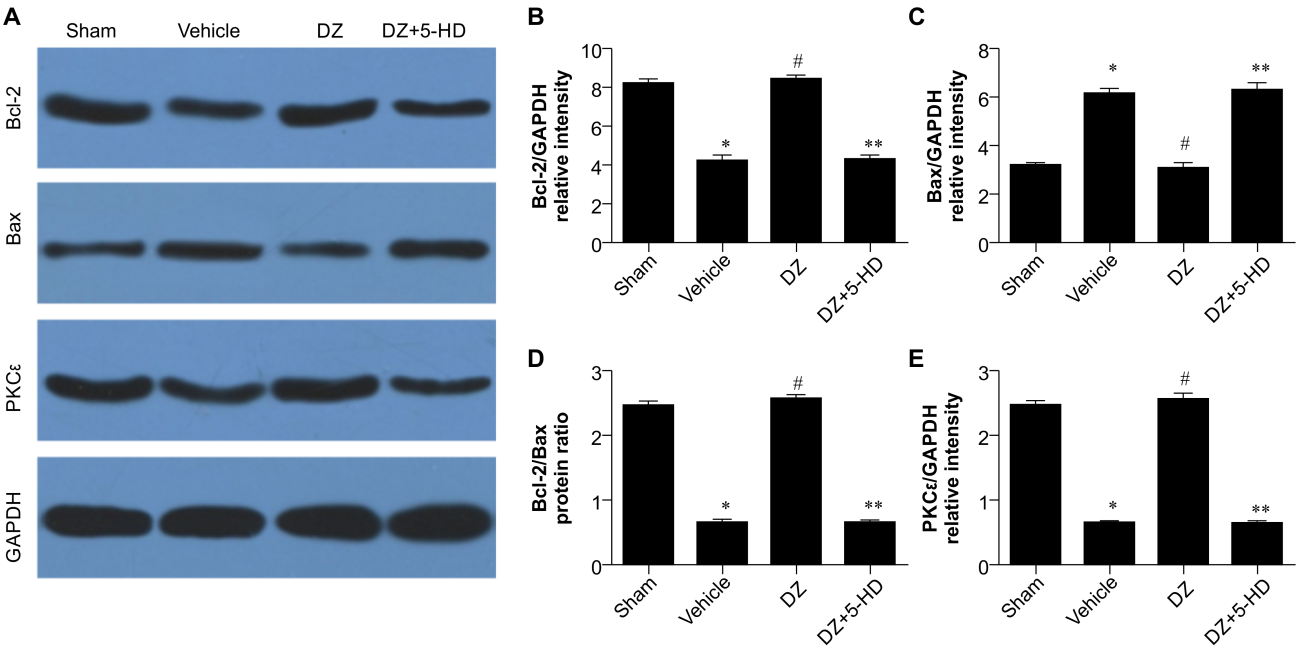


**Supplementary Figure 3.** Expression of Bcl-2, Bax, and PKCε protein following ROSC. Expression of Bcl-2, Bax, and PKCε protein in the cerebral cortex 24 h after ROSC in a rat model of cardiac arrest was assessed using Western blotting (A). Western blots were quantified by densitometry (B−E). Data are presented as means ± SD, n = 5 rats/group. **P <* 0.01 vs. sham group; ^#^*P <* 0.05 vs. vehicle group; ***P <* 0.05 vs. DZ group; ROSC: return of spontaneous circulation; GAPDH: Glyceraldehyde 3-phosphate dehydrogenase.


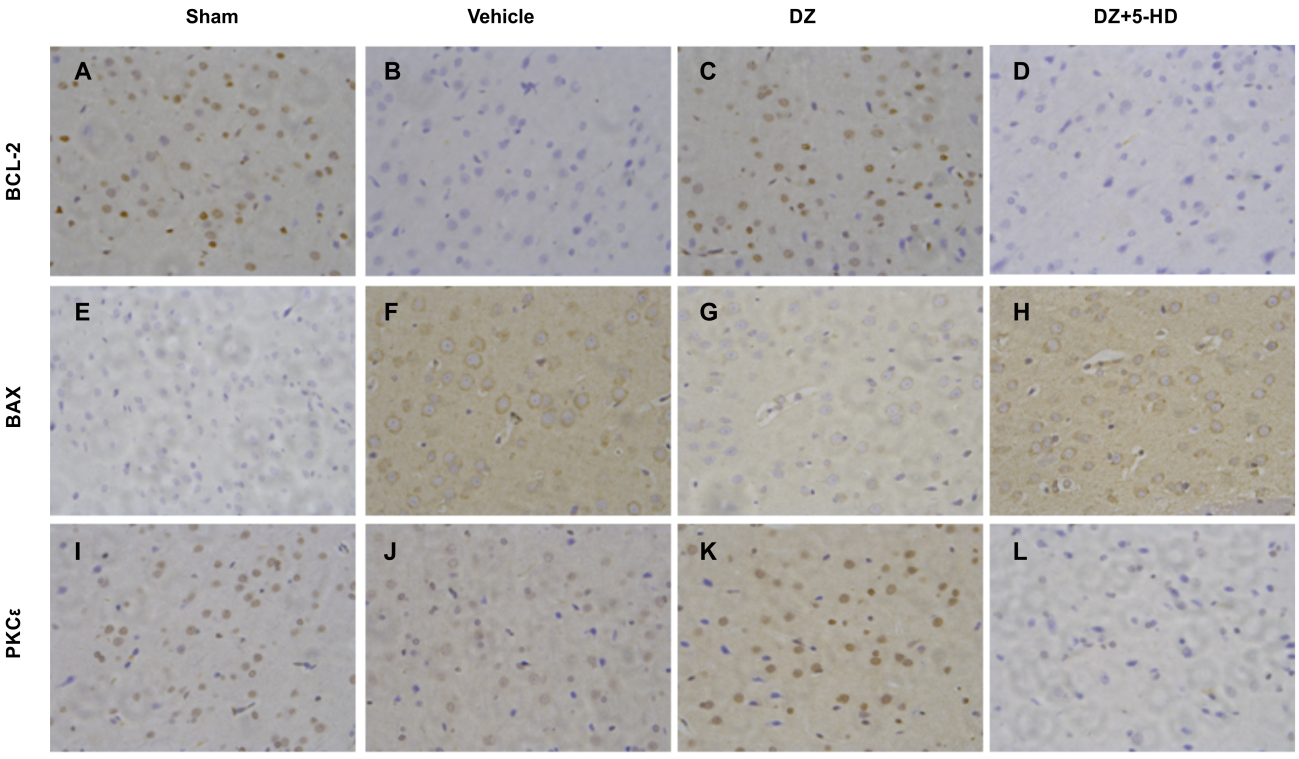


**Supplementary Figure 4.** Expression of Bcl-2, Bax, and PKCε protein in the cerebral cortex 24 h after ROSC in the four rat groups obtained via immunohistochemical staining. (A, E, I) Sham; (B, F, J) Vehicle; (C, G, K) DZ; (D, H, L) DZ+5-HD; ROSC: return of spontaneous circulation.
